# Neural Processing of Short-Term Recurrence in Songbird Vocal Communication

**DOI:** 10.1371/journal.pone.0011129

**Published:** 2010-06-17

**Authors:** Gabriël J. L. Beckers, Manfred Gahr

**Affiliations:** Department of Behavioural Neurobiology, Max Planck Institute for Ornithology, Seewiesen, Germany; Pennsylvania State University, United States of America

## Abstract

**Background:**

Many situations involving animal communication are dominated by recurring, stereotyped signals. How do receivers optimally distinguish between frequently recurring signals and novel ones? Cortical auditory systems are known to be pre-attentively sensitive to short-term delivery statistics of artificial stimuli, but it is unknown if this phenomenon extends to the level of behaviorally relevant delivery patterns, such as those used during communication.

**Methodology/Principal Findings:**

We recorded and analyzed complete auditory scenes of spontaneously communicating zebra finch (*Taeniopygia guttata*) pairs over a week-long period, and show that they can produce tens of thousands of short-range contact calls per day. Individual calls recur at time scales (median interval 1.5 s) matching those at which mammalian sensory systems are sensitive to recent stimulus history. Next, we presented to anesthetized birds sequences of frequently recurring calls interspersed with rare ones, and recorded, in parallel, action and local field potential responses in the medio-caudal auditory forebrain at 32 unique sites. Variation in call recurrence rate over natural ranges leads to widespread and significant modulation in strength of neural responses. Such modulation is highly call-specific in secondary auditory areas, but not in the main thalamo-recipient, primary auditory area.

**Conclusions/Significance:**

Our results support the hypothesis that pre-attentive neural sensitivity to short-term stimulus recurrence is involved in the analysis of auditory scenes at the level of delivery patterns of meaningful sounds. This may enable birds to efficiently and automatically distinguish frequently recurring vocalizations from other events in their auditory scene.

## Introduction

The ability to efficiently process frequently recurring vocal signals and distinguish them from novel ones may be an important adaptive trait to many group-living animals. Frequently experienced vocalizations do not normally require a new decision to be made with each recurrence, and the neural systems underlying neural processing of vocalizations may prevent the recruitment of costly cognitive resources, e.g. attention, required for processing new or unexpected events. Moreover, the information content of frequently recurring signals may be highly redundant. At the neural level, this could be exploited by using efficient sensory coding strategies leading to a reduction in metabolically expensive neural firing [1], while simultaneously enhancing discrimination [2], [3]. However, it remains unknown whether animals indeed process frequently recurring vocalizations differently from novel ones.

The auditory cortex is a key site where stimulus-specific sensitivity to short-term delivery of recurring sounds arises, both in humans [4] and other mammals [3], [5]–[7]. Pre-attentive sensitivity to stimulus recurrence occurs at time scales ranging from hundreds of milliseconds to tens of seconds, and has been suggested to underlie the fundamental processes in analyzing natural auditory scenes, such as in optimized coding, stream segregation, binding of auditory objects, or change detection in regular auditory input [8]. Most previous work utilized artificial stimuli and recurrence rates that do not reflect problems that perceptual systems need to solve in natural situations. It remains unclear whether or not neural sensitivity to short-term stimulus history underlies the analysis of natural delivery patterns of meaningful sound objects normally encountered during communication.

**Figure 1:**
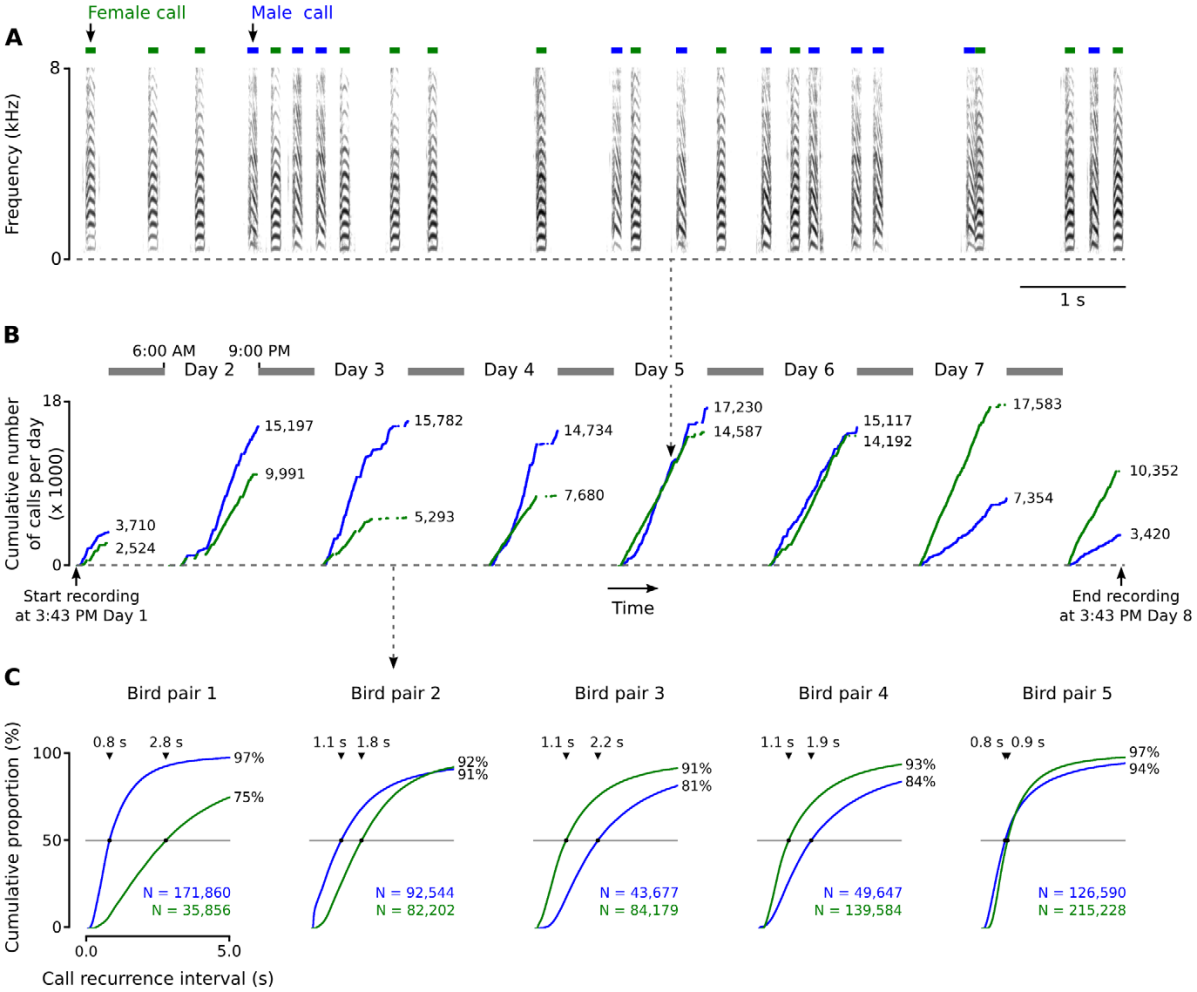
Short-range contact call delivery by a male–female pair of zebra finches. (A) A 10-s example of a communication scene. Green and blue bars indicate female and male calls respectively. (B) A mass-evolution graph [9] shows the cumulative number of calls per individual per day, against occurrence in time. Shown is one pair of birds (pair 2 in C). Shaded bars indicate night-time. (C) Cumulative distributions of the duration between two sequential short-range contact calls from one individual (‘call recurrence interval’) for all five pairs measured in this study. Triangular markers indicate the location and value of the median call interval for a specific bird.

Here we address the issue of neural sensitivity to stimulus history in the zebra finch, *Taeniopygia guttata*, a social bird that maintains communication with group-members using frequently repeated short-range contact calls (Figure 1A). We first set out to record and analyze complete auditory scenes of spontaneously communicating zebra finch pairs over week-long periods, and show that the time scales at which short-range contact calls recur match those at which sensory systems in mammals are sensitive to recently encountered stimuli. Next, we recorded neural responses in the auditory forebrain of anesthetized zebra finches to sequences of calls delivered at a range of rates that fit natural behavior. The results show that not only pre-attentive sensitivity to call delivery dynamics exists in the avian auditory forebrain, but also that this process is widespread across functionally different areas. Furthermore, the amount of neural firing at typical call recurrence rates is significantly reduced with respect to slow rates, suggesting that short-term plasticity of these neural systems contributes to efficient processing of natural communication behavior. Lastly, we show that recurrence-dependent modulation of neural responses is call-specific in secondary centers of the auditory forebrain, where it may pre-attentively underlie the ability to distinguish between common and rare vocalizations.

## Results

### Calling behavior

All vocal behavior from five pairs of zebra finches was recorded in an acoustically isolated laboratory environment for approximately one week each (mean 7.6 days; range 6.1–10.8 days). This dataset, consisting of more than one million call events, revealed that short-range contact calling in zebra finches is a very common behavior. The mean number of identified calls per bird per day was 14,095 (range: 4,044–28,036) for males and 15,215 (range: 5,849–30,747) for females. Mass evolution graphs [9] of calling behavior by each bird confirm earlier anecdotal observations [10] that zebra finches nearly continually produce these short-range signals during the day (Figure 1B). We quantified call recurrence intervals by calculating the time difference between the onset of each call and the onset of the subsequent call produced by the same animal. Figure 1C shows cumulative distributions and mean duration of these intervals for each bird. The median short-range contact call recurrence interval is on average 1.4 s (range 0.8–2.2 s) for male birds and 1.5 s for females (range 0.9–2.8 s).

### Neural responses to recurrent calls

**Figure 2:**
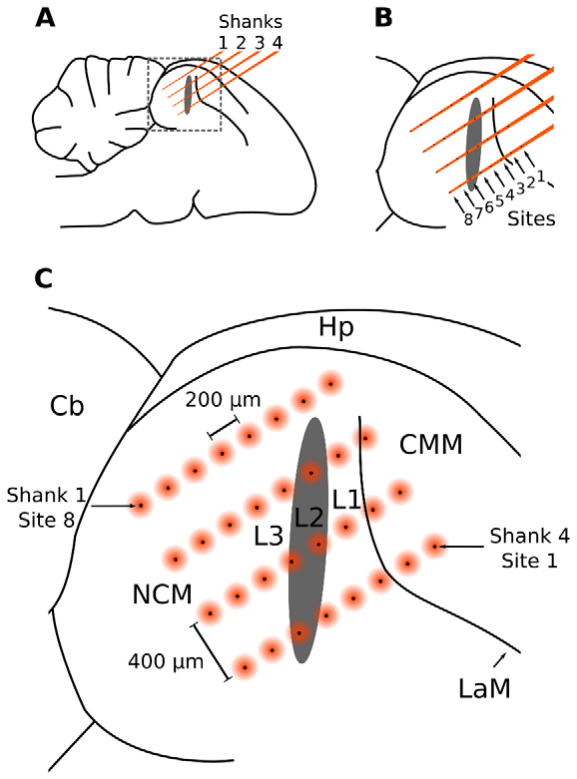
Schematic representation of the silicon multi-electrode array situated inside the auditory forebrain. (A) The four equidistant and parallel shanks of the array are situated in a parasagittal plane in the medio-caudal forebrain. (B) Each shank contains eight electrodes (‘sites’). (C) The matrix of 32 sites cover a relatively large area from which neural responses can be recorded in parallel, including the anatomical Field L, consisting of subfields L1, L2, and L3, and NCM and CMM. The black spots represent electrode sites, while the orange circles indicate that recorded potentials may originate from a field around these sites. Hp: Hippocampus, Cb: Cerebellum, NCM: caudomedial nidopallium, CMM: caudomedial mesopallium, L1, L2, L3: subdivisions of Field L; LaM, lamina mesopallialis [11], [34].

Next we tested the sensitivity of auditory systems to call delivery dynamics by recording the neural responses to sequences of call stimuli differing in their recurrence statistics. Because the auditory cortex is most likely the key site where short-term stimulus-specific and probability-dependent auditory responses are generated in mammals [4], [3], [5], [6], we directed our recordings from within the medio-caudal auditory forebrain (Figure 2). This part of the brain contains a sheet-like structure, L2 [11]–[13], the main thalamo-recipient auditory forebrain station, which is thought to be the avian analog of the mammalian layer IV of primary auditory cortex; we therefore refer to L2 as ‘primary auditory area’. L2 projects to the surrounding auditory structures L, L1, L3, caudomedial nidopallium (NCM) and caudomedial mesopallium (CMM); which receive only sparse thalamic input and which we collectively refer to as ‘secondary auditory areas’. Some secondary areas have been compared to mammalian auditory association cortex [14]. Neurons from these areas respond to many classes of both artificial and natural sound [15]–[26], but their sensitivity to short-term recurrence of artificial or natural stimuli has not been previously addressed.

**Figure 3:**
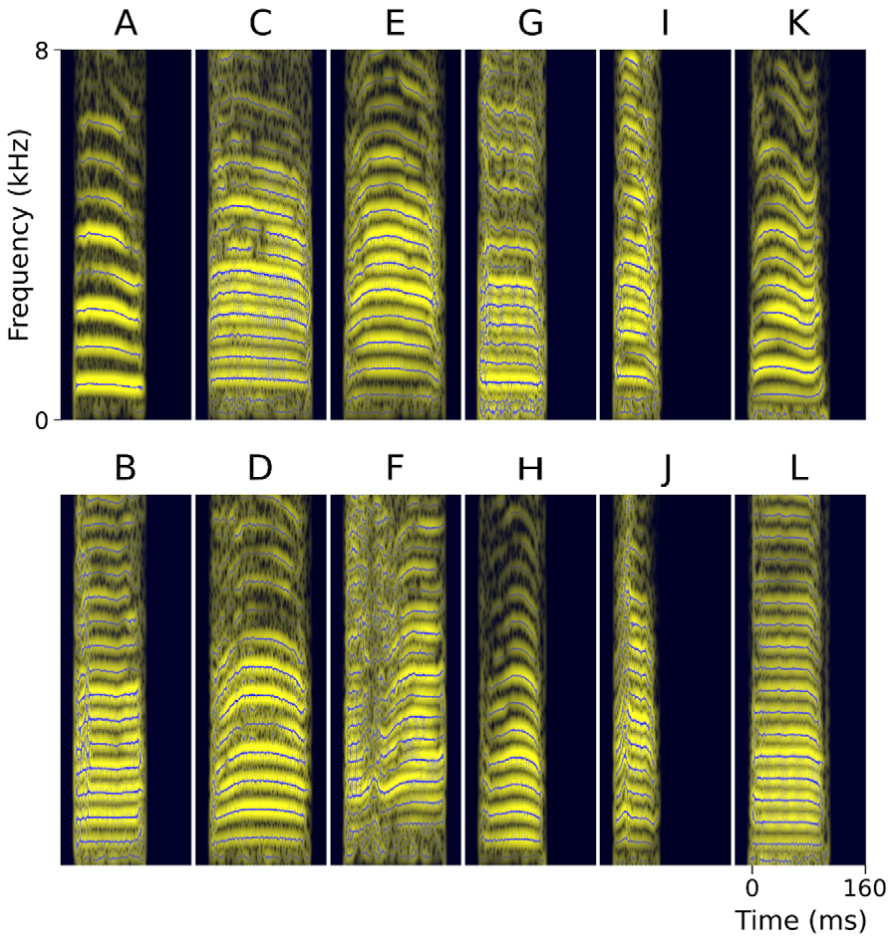
Spectrographic representation of the 12 male zebra-finch short-range contact calls used as stimuli in this study. Calls presented in columns are matched for duration. Shown are spectrograms (light bands) that have been calculated with a short-time Fourier transform, superimposed with a reassignment-based sparse time-frequency representation (dark lines; settings: 23 ms Gaussian analysis window, consensus of σ range 0.8–3.5, 0.25 ms step duration, 25 dB dynamic range; [55]). Call parameters (duration/mean fundamental frequency): A: 89 ms/784 Hz, B: 89 ms/528 Hz, C: 128 ms/452 Hz, D: 128 ms/591 Hz, E: 127 ms/551 Hz, F: 127 ms/433 Hz, G: 83 ms/413 Hz, H: 83 ms/570 Hz, I: 57 ms/470 Hz, J: 57 ms/530 Hz, K: 101 ms/564 Hz, L: 101 ms/470 Hz. Fundamental frequency was determined with an autocorrelation algorithm [48].

Based on the analysis of spontaneous calling behavior, we decided to use six sequences with stimulus delivery intervals of 5000, 2500, 1250, 625, 313, and 156 ms. Each sequence consisted of two calls originating from different male individuals (Figure 3) and were randomly intermixed: one was presented 900 times (‘common call’) and the other one 100 times (‘rare call’). In this design, common call delivery models the recurrence dynamics of short-range contact calling in communication scenes. Rare calls are not intended to model a particular natural behavior, but are used as a probe stimulus to test whether potential neural sensitivity to call recurrence dynamics is call-specific. We assigned a different common-rare call pair to each of the six sequences presented to each bird, balanced the assignment of call pairs to different sequences across birds, and switched the common/rare roles of calls within pairs in half of the birds.

We played the six different stimulus sequences in random order to 12 anesthetized female zebra finches while recording neural responses with high-density silicon multi-electrode arrays [27], [28] (Figure 2). This technique enabled us to record analog multi-unit action potentials (AMUA) and local field potentials (LFP) from a matrix of 32 electrode sites in parallel. AMUA signals reflect action potential activity of relatively small neuronal populations near the recording site [29], and LFP signals reflect coordinated post-synaptic activity of larger groups of neurons that may be situated further away [30]. AMUA signals allow for more precise localization of responses, while LFP signals have the advantage of including sub-threshold input.

**Figure 4:**
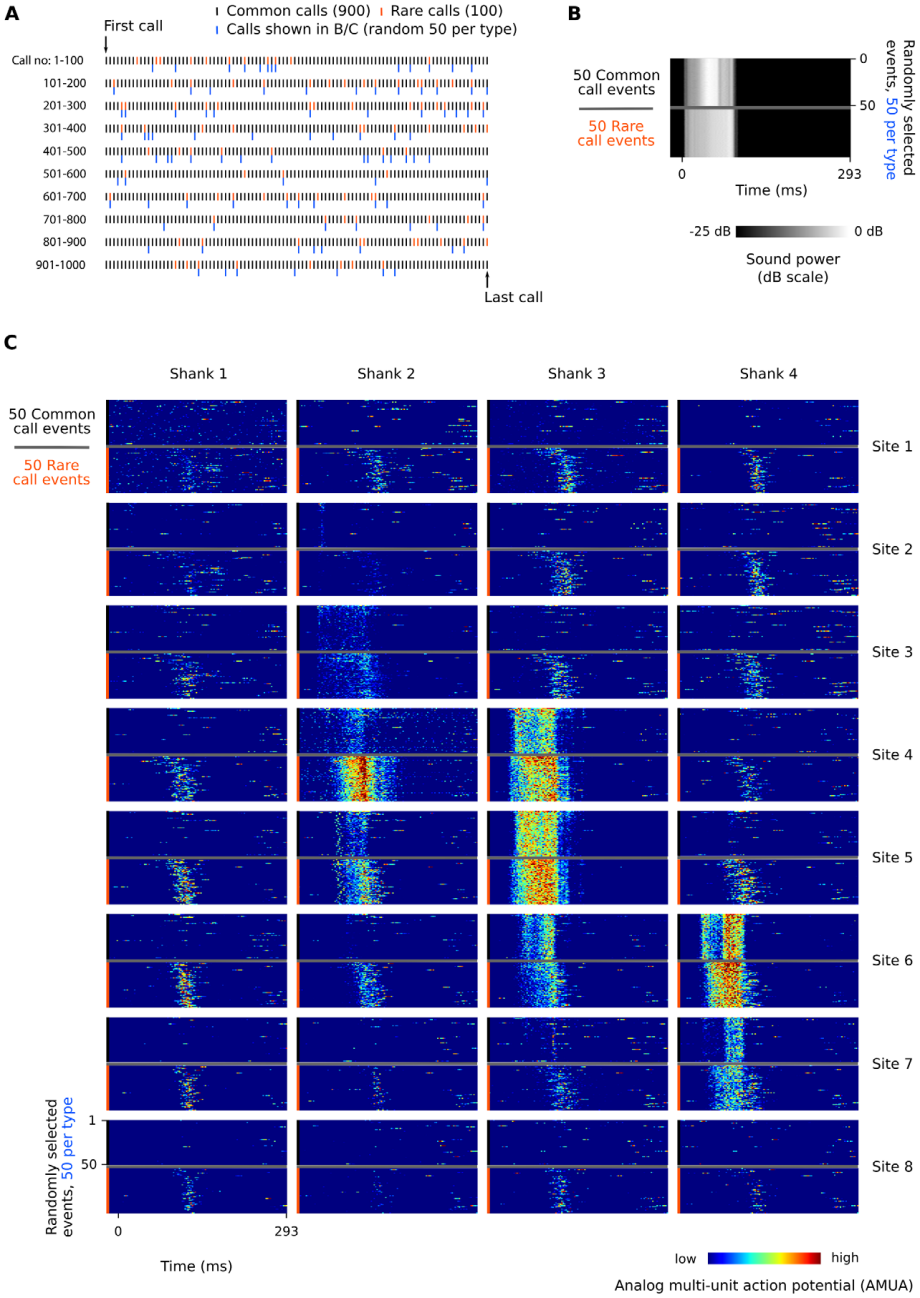
Example of AMUA responses to common and rare calls in the 625 ms sequence (Bird 3, calls K and L, respectively). (A) Common calls (900, black marks) are randomly interspersed with rare calls (100, orange marks). Blue marks indicate the 100 calls (50 per type) that have been randomly selected to be shown in subfigures B and C. (B) Call stimuli are recorded synchronously with the electrophysiological signals to verify correct alignment of measurement episodes in our analyses. (C) Raster plots of AMUA signals in response to randomly selected sets of 50 common calls and 50 rare calls. Common and rare calls are shown separately although they have been presented to the bird in a random mixture (see A). Color represents AMUA amplitude, scaled per site, and clipped to 25% and 75% of the total signal range for visual presentation only.

We first inspected raster plots of all call responses (Figure 4) to get an overview of how neural response patterns to recurring call stimuli are distributed spatially over the auditory forebrain. From these, two types of response patterns are apparent.

**Figure 5:**
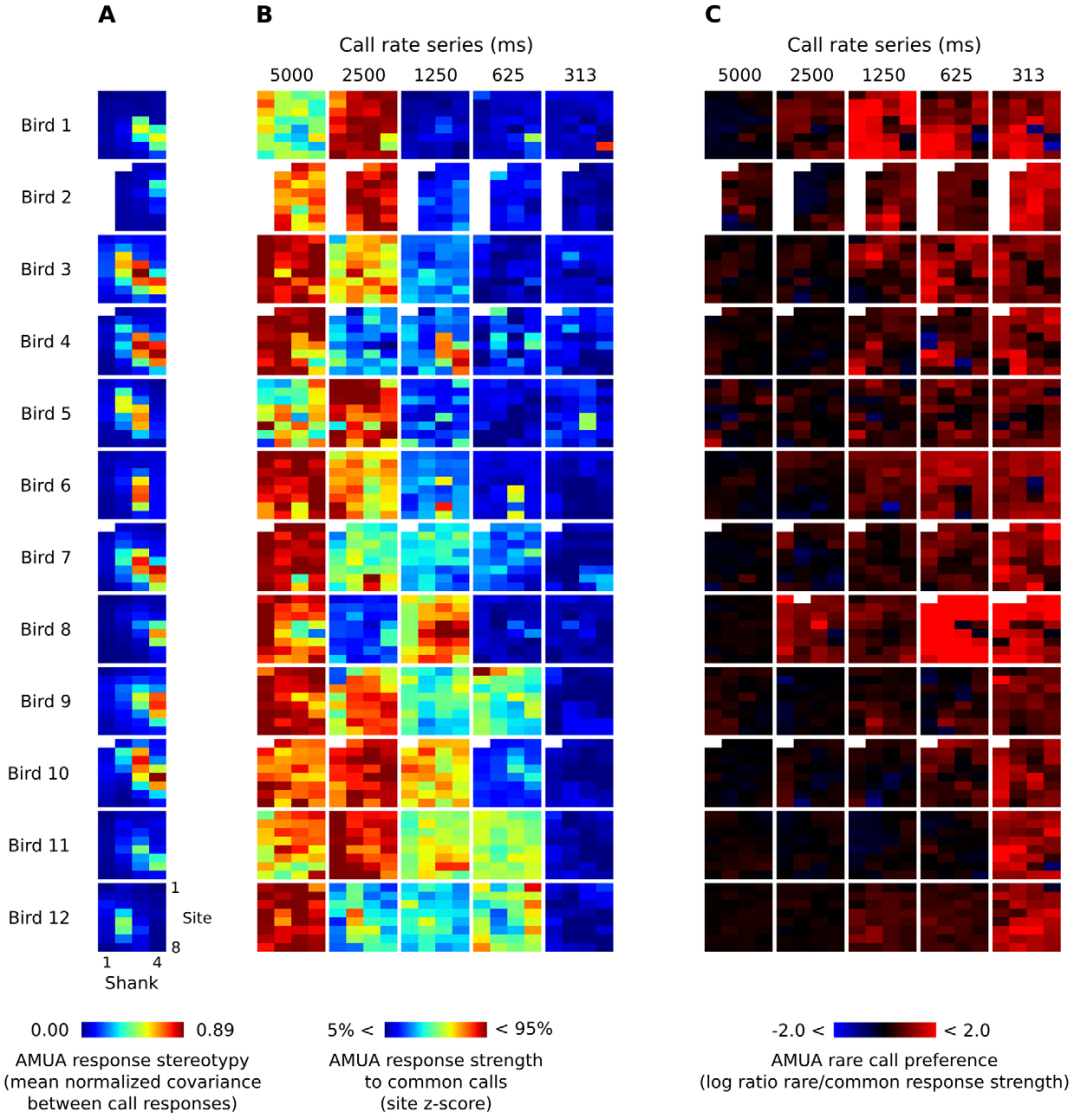
AMUA response measures for all sites from which we recorded in this study. Each of the 32-pixel colored square corresponds to the matrix of 4×8 sites of a multi-electrode array that was used for simultaneous recordings (see Figure 2). (A) Response stereotypy for each electrode site in each bird, based on responses to both common and rare calls in all six sequences. Sites with a relatively high response stereotypy correspond to the anatomical area L2. In four birds one or more sites are lacking, because they did not show auditory AMUA responses. (B) Modulation of response strength to common calls between call series with different recurrence rates. Response strength has been normalized to a *z*-score per site; color differences between sites are thus meaningless. Scores outside the 5–95% range have been clipped for visual presentation. Note that for each series birds received different calls, which may explain part of the variation in response strength. (C) Rare call preference, calculated as the log of the ratio between mean response strength to rare and common calls within one series. A score of 0 (black sites) indicates no preference, while negative scores (blue sites) indicate a common call preference and positive scores (red sites) indicate a rare call preference. Scores have been clipped to range between −2 and 2 for visual presentation.

In a minority of the 32 recorded sites in each bird, AMUA responses occurred almost exclusively during the call stimulus after taking into account a ∼10 ms time delay for the information to reach the forebrain. These responses are stimulus-locked and are strongly stereotypic in their firing pattern (see, e.g., sites 4 and 5 on shank 3 in Figure 4C). We quantified the level of stereotypy in AMUA response patterns for each site by calculating the mean normalized covariance between all response signals per call, starting at the beginning of a call and ending at the start of the next call, and using the mean value as an index for response stereotypy. Inspection of the spatial distribution of response stereotypy indices revealed that sites with a high index of response stereotypy cluster together in an oblong shape (Figure 5A). Post-hoc histological analysis of the electrode tracts showed that these clusters correspond to L2. Given that L2 is morphologically distinguishable [11] and that this area shows distinct, quantifiable response patterns, we used the spatial distribution of response stereotypy indices as a functional map to indicate the position and orientation of the electrode array in the auditory forebrain with respect to this subarea in each bird.

**Figure 6:**
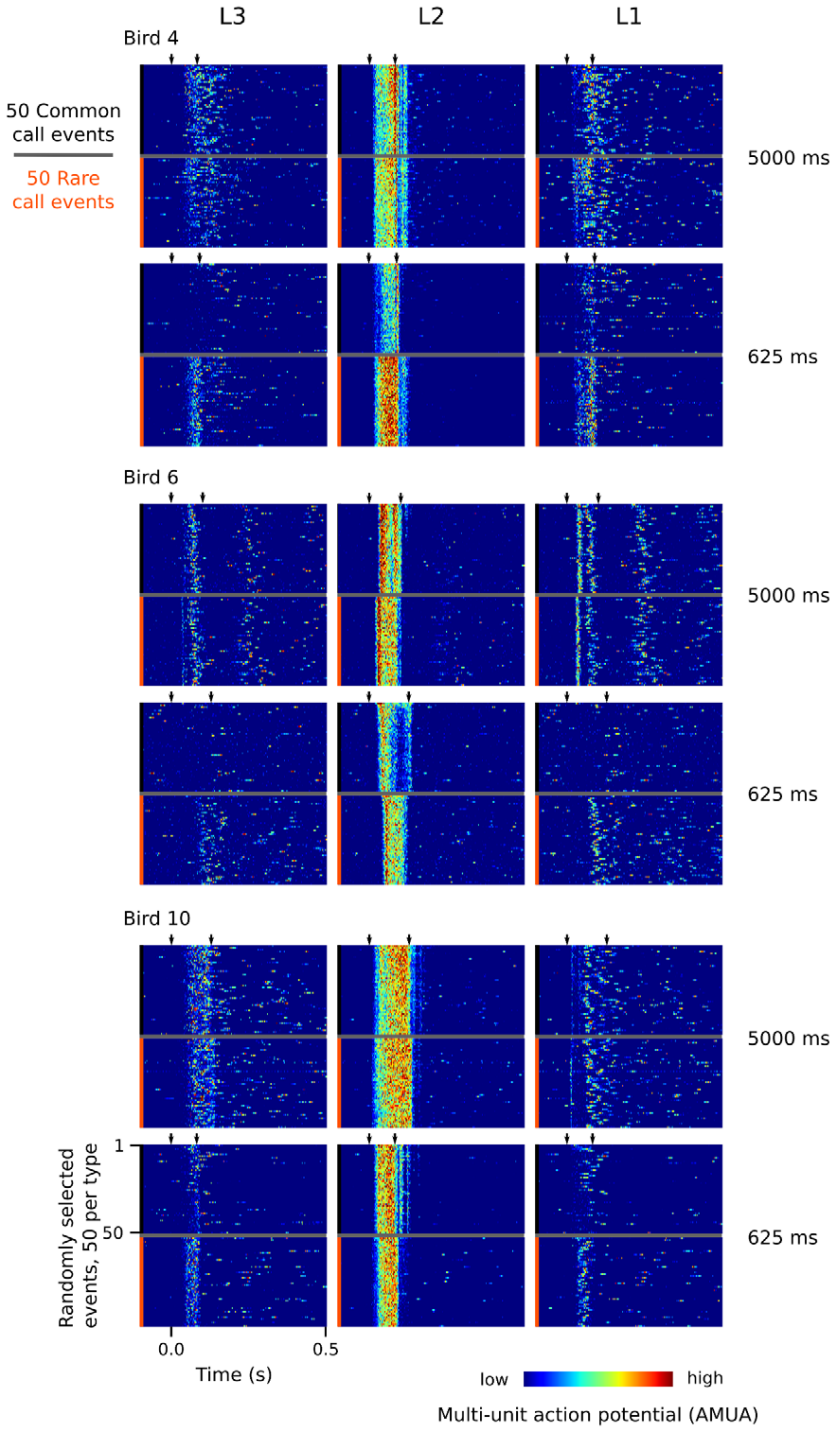
Examples of response patterns to a random 50 common and a random 50 rare calls in three different birds for two different rate sequences. For each bird and rate sequence, the responses of three sites are shown: the middle column shows that of a L2 site, while the first and last column show that of adjacent L3 and L1 sites, which are situated caudal and rostral to L2, respectively. The sites (shank, site) shown are: Bird 4 (2,6), (3,5), (4,4), Bird 6: (2,6), (3,4), (4,5), Bird 10: (3,8), (4,5), (4,3). Color represents AMUA amplitude, scaled per site, and clipped to 25% and 75% of the total signal range for visual presentation only.

The remaining sites, surrounding L2, responded to recurring calls with brief bursts of activity that varied in latency from call to call, characterized by low response stereotypy indices (Figure 5A). Moreover, stimulus history appears to change the activity patterns of these neurons in a different manner: common call events at faster rates often completely lack the typical response patterns visible at lower rates. Figure 6 provides additional examples of the distinct differences in response patterns between sites in L2 and those in the immediately adjacent secondary auditory areas. These examples also show that call-related responses in secondary areas may continue long after the sound of the call stimulus has finished, as has been described earlier for responses to pure tones in NCM [31].

### Auditory responses are modulated in response to call-specific delivery statistics

To test whether or not short-term statistics in the delivery of calls are important to their neural processing, we compared response strengths of rare and common calls across sequences, differing in their call delivery rate. This comparison must be based on a fixed response measurement interval, limited by the minimum interval between two calls in the fastest sequence. Raster plots of our recordings show that responses can outlast the call stimulus and that including the 156 ms sequence would limit us to the use of an unreasonably short measurement interval for the five slower sequences. Therefore we excluded the 156 ms sequence from this analysis, enabling us to increase the measurement interval to 293 ms (313 ms mean interval minus a maximum of 20 ms deterministic jitter, see Materials and Methods). This decision did not influence the outcome of the statistical tests reported in this paper (see below).

First we explored the patterns of AMUA response strength modulation as a function of both call delivery rate and spatial location in the auditory forebrain (Figure 5B). This revealed that in the great majority of sites, response strengths to common calls decrease as calling rates increase. For the sequences of 2500, 1250, 625, and 313 ms, the percentage of sites (*N* = 372) that show a decrease in response level in comparison to the 5000 ms sequence is 63%, 91%, 98%, and 99%, respectively. Because recording sites are distributed spatially over a relatively large area, spanning multiple anatomical fields (Figure 2), these results show that modulation of response strength due to call recurrence statistics is a widespread phenomenon within the auditory forebrain. Note that for each sequence, birds received a new pair of calls that differ in many acoustic features from those in the other sequences (Figure 3). This may explain part of the variation in response strength between sequences within birds; however, the assignment of call pairs between birds was counterbalanced across the different sequences.

The processes underlying delivery rate-dependent modulation of neural responses to recurring calls shown in Figure 5B could be stimulus specific or acting more generally on auditory input. To distinguish between these two explanations, we compared response strength of common calls to that of rare calls by calculating the ratio of mean response strength of rare calls to that of common calls. Data were expressed on a log scale and were designated as ‘rare call preference’. If modulation of responses is not call specific, or call specific at a small minority of sites, then the overall the number of sites with a positive rare call preference should not deviate significantly from the number of sites with a negative rare call preference, however this is not the case (Figure 5C). The percentage of sites (*N* = 372) responding more strongly to rare calls than to common ones is 63%, 65%, 86%, 86%, and 95%, for the 5000, 2500, 1250, 625, and 313 ms sequences, respectively. Overall, the level of rare call preference increases as recurrence rate increases, although sites with a high stereotypy index (Figure 5A) do not seem to follow this general pattern (Figure 5C).

**Figure 7:**
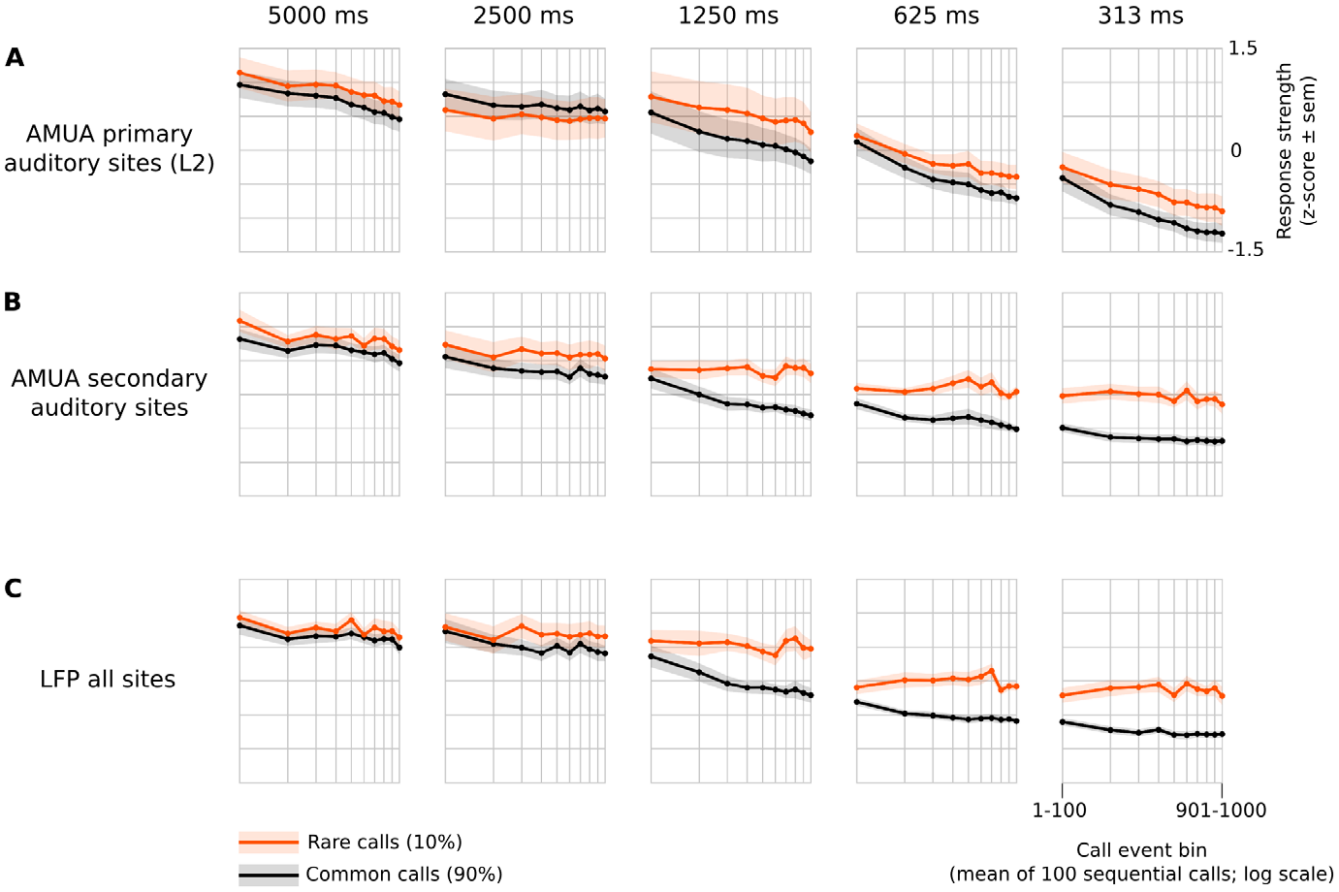
Response strengths decrease with calling rate and are partly stimulus-specific. The responses are standardized (*z*-scores), but note that all statistical tests in this study are based on absolute response levels. Shown are the mean of these values over birds (± standard error of the mean as shaded color), binned per 100 sequential call events and split between common and rare calls. (A) Mean (*N* = 9 birds) AMUA response strength at primary auditory sites. These sites have been classified as ‘primary’ based on their stimulus-locked, stereotypic response characteristics only; such sites cluster in a shape that corresponds to the anatomical area L2. (B) Mean (*N* = 12 birds) AMUA response strength at secondary auditory sites, i.e. sites whose auditory responses are not stereotypic responses and that surround L2 (i.e. L1, L3, NCM and CMM). (C) Mean (*N* = 9 birds) LFP response strength, which is not split between primary and secondary sites because local field potentials may not originate from the immediate vicinity of the site at which they are recorded.

To get an overview of how site activity is modulated by call recurrence rate and probability (common/rare), we pooled sites into two groups consisting of those with high response stereotypy (>0.5; ‘primary auditory sites’) and those with low response stereotypy (<0.5; ‘secondary auditory sites’), and averaged the absolute response strengths within these groups in 100-call sequence bins per bird (Figure 7). This shows that, overall, AMUA activity is reduced for both common and rare calls when delivery rates are increased (Figure 7A, B). At primary auditory sites (Figure 7A), the difference between responses to rare and common calls at a given rate is insignificant, showing that response modulation due to recurrence is independent from the recurring call. By contrast, AMUA responses to rare calls are much stronger than to common calls at faster rates (≤1250 ms) at secondary auditory sites (Figure 7B). This shows that recurrence-dependent response modulation is in part call-specific at secondary auditory sites. However, there is also a non-specific effect, because responses to rare calls in the 1250 ms and 625 ms series are weaker than responses to common calls in the 5000 ms series (Figure 7B), even though their average recurrence rate is lower. The same holds true if one compares responses to rare calls in the 313 ms series to that of the common call in the 2500 ms series (Figure 7B).

LFP responses (Figure 7C), which are less local than AMUA responses and, consequently, were not split into groups of different site types, are similar to AMUA responses at secondary auditory sites (Figure 7B).

A linear mixed-model regression of *recurrence rate*, *probability* and *site type* on AMUA response strength shows that all three variables and their interactions are statistically significant factors in explaining the observed variation (Table 1), and therefore confirms the patterns described above. Note that there is also a two-way interaction between the factors *site type* and *probability*, and a three-way interaction between *calling rate*, *site type*, and *probability*, demonstrating a significant difference in the call-specificity of recurrence-dependent modulation between primary and secondary auditory sites (Figure 7A vs 7B). A separate model encompassing only primary auditory sites does not show a significant effect of *probability* (*t* = 0.81; *p* = 0.20) or an interaction of *probability* and *recurrence rate* (*t* = 1.30; *p* = 0.42), suggesting that the recurrence-dependent modulation of response strength is either not call-specific, or is so weak that it cannot be detected with the current experimental design. In a separate model of the group of secondary auditory sites, in contrast, these factors are highly significant (*t* = 7.17; *p<*0.0001, and *t* = 3.89; *p* = 0.0002, respectively), showing that in secondary auditory areas such modulation is call-specific. A linear mixed-model regression of LFP responses shows that all factors and their interactions are statistically significant (Table 1). Rerunning the statistical models using the much shorter response interval of 136 ms and including the 156-ms sequence led only to minor changes in the reported *t-* and *p*-values.

**Table 1:** Significance of factors that explain neural response strength in a linear mixed regression model.

Signal Type	Factor^a^	*t*-Value	*p*-Value
**AMUA**	Calling rate	7.05	<0.0001
	Site type (primary/secondary auditory area)	10.65	<0.0001
	Probability (Common/rare)	7.47	<0.0001
	Calling rate : Site type	2.90	0.0041
	Calling rate : Probability	4.05	0.0001
	Site type : Probability	4.07	0.0001
	Calling rate : Site type : Probability	2.15	0.0330
**LFP**	Calling rate	6.43	<0.0001
	Probability (Common/rare)	6.49	<0.0001
	Calling rate : Probability	3.59	0.0005

a interactions are denoted with “:”.

The significant differences in response between the different recurrence rate series show that the mechanisms underlying stimulus-history dependent processing must include responses that are sensitive over short time scales, i.e. hundreds of milliseconds to multiple seconds. Indeed, the response measurements in Figure 7, which are binned per 100 sequential calls, show that at fast delivery rates (intervals ≤625 ms) a major component of the response modulation is due to recurrences already established within the first 100 calls. Longer integration windows, which have been shown to be involved in neural adaptation to tone stimuli in cat primary auditory cortex [8], may also play a role, especially at primary auditory sites where a decrease in response strength continues even after hundreds of calls. The experimental design of the current study, however, is not suited to directly address this issue.

What is the total amount by which electrical signaling is modulated as a result of call recurrence rate? For the sequences 2500 ms, 1250 ms, 625 ms, and 313 ms, the total amount of AMUA activity per common call decreases (mean ± SEM; *N* = 12) to 94% (±11%), 64% (±7%), 45% (±4%), and 27% (±3%), respectively, compared to those of the 5000 ms sequence. It might be argued that these values overestimate the real effect because AMUA signals are subjected to thresholding in order to exclude background noise (fixed per bird, see Materials and Methods). Nevertheless, the same calculation applied to the total amount of LFP activity, which is simply the mean power of raw brain signals between 0.1 and 350 Hz without any further conditioning, shows a similarly large effect. For the sequences 2500 ms, 1250 ms, 625 ms, and 313 ms, activity is reduced to 102% (±11%), 66% (±7%), 35% (±5%) and 21% (±2%), respectively, compared to that of the 5000 ms sequence.

**Figure 8:**
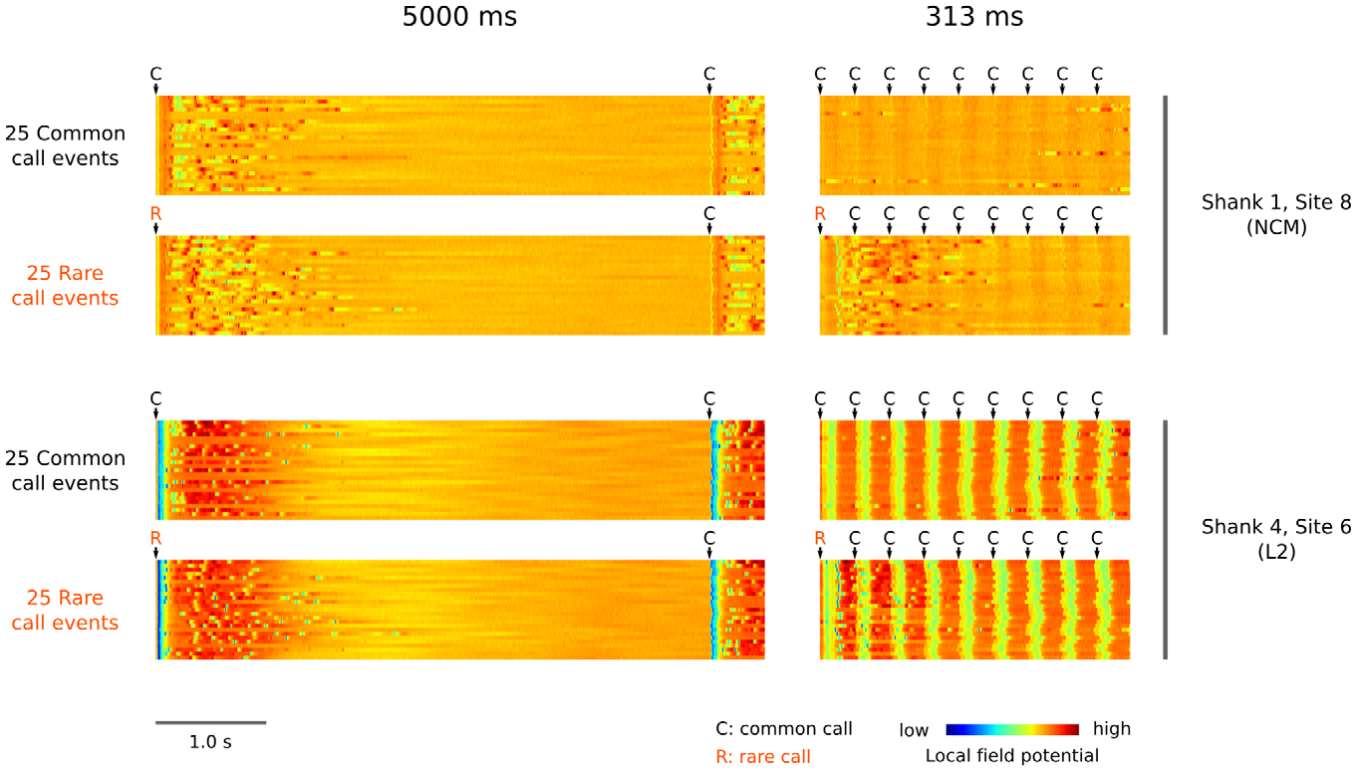
Call-event related responses can last for a long time after a call has finished and responses of calls may overlap. This is shown here using an example of LFP recordings in bird 1 at two different sites (NCM: top two rows, L2 bottom two rows) and two different recurrence rates (left column: 5000 ms series, right column 313 ms series). In the slow 5000 ms series, LFP responses to both common calls (random 25 events) and rare calls (random 25 events) can be seen to last up to seconds after the call event in both brain areas. In the fast 313 ms series, responses to common calls in the NCM site are almost completely absent, while those to rare calls are still visible. In the L2 site, responses to common calls have not disappeared but are clearly reduced. Importantly, in the 313 ms rate series responses to rare calls can be seen to continue during the presentation of a sequence of four subsequent common calls. Note that the actual common and odd call stimuli in the 5000 ms and 313 ms series are different (I/J and C/D of Figure 3, respectively). The jitter that is visible in the responses in L2 to subsequent calls, relative to the first one, is due to a small amount of deterministic jitter that we applied to the delivery of stimuli (see Materials and Methods).

Given the strong differences in response strength between common and rare calls at faster calling rates, it may seem profitable to single out specific sequences of calls for separate analysis [8], [32]. For example, a comparison between response strengths to common calls that followed a rare call and that of common calls that followed another common call might reveal a ‘dishabituation effect’. However such comparisons are based on the assumption that responses do not overlap in time, and in our data set responses in secondary auditory areas may continue long after a call has finished. In fact we have observed that responses to rare calls appear to overlap with those to following common calls. Figure 8 shows an example of this phenomenon at both a primary and secondary auditory site: at a fast recurrence rate (313 ms series, right column) there is activity during common calls that follow a rare call which is absent during common calls that do not follow a rare call. A comparison with the response patterns at slow recurrence rates (5000 ms series, left column) suggest that this activity is caused by late response components of the rare call, and not by responses to the common calls that follow the rare call. This interesting phenomenon complicates the interpretation of ‘response strength’ to a specific call event, because it is not generally clear how much of the response strength is due to the focal call and how much is due to earlier events. However, analyses of specific sequences are not necessary to answer the main question of the current study, because common and rare calls in our experiment have the same probabilities of being preceded by a given call sequence. Statistical differences between rare and common calls during the 293-ms ‘response’ interval must thus be caused by their different recurrence statistics.

## Discussion

Many animal communication scenes, especially those of group-living animals such as zebra finches, are rife with recurring vocalizations, but this phenomenon has remained poorly studied at the quantitative level. In the current study we first showed that individual zebra finches in a laboratory setting produce up to tens of thousands of short-range contact calls per day, with median call intervals of about 1.5 s. This finding corroborates earlier qualitative reports of wild zebra finch auditory scenes, which are described as containing a continuous ‘background’ of such sounds [10]. To zebra finches and other group-living birds, however, short-range contact calls do not constitute background sound but rather on-going communicative behavior that needs to be monitored in order to increase survival [10], [33]. How are the neural systems that analyze such active communication scenes able to do so efficiently? Two of our findings are important in this respect.

First, we found significant differences in the neural response strength elicited by rare and common calls in secondary auditory areas even though birds were anesthetized, demonstrating that sensitivity to short-term delivery rates of specific vocalizations is already established pre-attentively, perhaps to prevent potentially expensive cognitive resources such as attention from being preoccupied with very frequent and predictable communicative events. The fact that responses to common calls often disappear at higher delivery rates fits with this hypothesis. ‘Automatic monitoring’ of frequently recurring calls may also make sense from a behavioral point of view because such communicative signals normally do not require new decisions to be made. Differences in response strength between rare and common calls are significant in secondary auditory areas, but not in the primary auditory area L2. Sensitivity to short-term recurrence of specific vocalizations may thus arise within secondary areas, which receive most of their input from L2 and from each other [34], or in the connectivity between L2 and secondary areas. Also, we found that the difference in response strength between rare and common calls is highly dependent on the rate of call recurrence: at a relatively slow rate of one call per 5000 ms there is no difference, while at 1250 ms, which is close to typical rates in natural behavior, and at faster rates, the differences are large, suggesting that the mechanisms underlying these differences are based on short-term auditory plasticity operating at a time scale from hundreds of milliseconds to multiple seconds. Within this range, recent calls may be more strongly represented in this form of memory and therefore elicit a weaker response when they recur. The overall increase of the response difference between common and rare calls with decreasing call intervals may then be due to a disproportionally large reduction in the call-specific neural response component of the common call with respect to the rare call, the average recurrence interval of which lies outside or at the extreme end of this time scale.

The second finding that suggests that sensitivity to recurrence at short time-scales contributes to efficient analysis of communication scenes is that the overall response strength in L2 and secondary auditory areas to calls decreases as their rate of recurrence increases. Action potential firing and local field potential power in response to common calls at one call per 1250 ms, similar to typical rates in communication, are down to about 64% and 66% of the levels at 5000 ms, respectively. For faster rates of one call per 313 ms, responses are reduced to 27% and 21% of 5000 ms levels, rendering support for the idea that call rate-dependent modulation of neural responses may lead to an overall reduction in energetic expenditure on communication.

One of the best known experience-related neural dynamics is a modulation of activity when sensory stimuli are repeated, most often a suppression [35]. In mammals such effects have specifically been shown for auditory cortex [36]–[39], [3], [8], [6]. However, these observations are based on paradigms with artificial and simple stimuli like pure tones. As a consequence, it is difficult to predict at what level of natural auditory scene analysis the observed phenomena are normally involved (e.g. stimulus coding, stream segregation, object binding or analysis of object delivery statistics), and thus what problems they solve. The current study is, to our knowledge, the first to show that the strength of forebrain auditory responses to conspecific vocalizations depends on the rate at which these signals are experienced within natural ranges, suggesting that the function of neural sensitivity to short-term stimulus delivery statistics includes the analysis of natural auditory scenes at the level of delivery patterns in communication.

The hypothesis we tested was that the auditory forebrain system in the zebra finch processes vocalizations differently depending on their short-term history. Our experimental design was optimized to establish such an effect by recording AMUA and LFP signals from many sites in parallel, while at the same time optimizing the external validity of the study by using a full suite of natural stimuli. Our design, however, is not optimized for addressing questions about the underlying mechanisms responsible for the differences in neural response strength between different call rates or probabilities. Indeed, because of our ‘systems level’ approach it is likely that the neural response strengths used as the measurement variable for statistical analyses is a reflection of multiple different neural processes sensitive to stimulus history.

In general, differences in neural response strength between rare and common sounds may be explained by two non-mutually exclusive hypotheses [40], [5]. The ‘adaptation hypothesis’ holds that responses to stimuli are suppressed when they recur [41], [42]. The difference in response strength between rare and common stimuli is determined by their recurrence rates, and thus the relative suppression of responses is different. An example of this can be observed in the N1 response in EEG recordings in humans, which shows rate-dependent attenuation when sounds are presented in sequences. The ‘model-adjustment hypothesis’, in contrast, holds that responses to rare sounds are stronger because they violate predictions produced by the neural representation of regularities in the repetitive stream of common sounds. In other words, the generation of the response is based on generative models in the brain which code auditory information and produce predictions of which sounds are likely to occur in the near future. For example, rare sounds that break the regularity of an auditory sequence elicit a pre-attentive EEG response in humans called mismatch negativity (MMN [4]), and can be interpreted similarly [43], [44]. In the current study, we did not address the question of which hypothesis may apply to the differences in auditory responses to rare and common calls in zebra finches. We used rare calls only as a control to test whether or not recurrence rate-dependent modulation of responses to common calls is call-specific and not to study the effects of stimulus novelty. The finding that overall response strength decreases for both common and rare calls as their delivery rate increases is in line with the adaptation hypothesis, but does not rule out the involvement of predictive processes. Also, a decrease in response strength is not necessarily caused by adaptation. In this light it is interesting to note that we observed two different AMUA response types to recurring calls. One type, wholly or mostly restricted to L2, is stereotyped and highly stimulus-locked. Delivery-dependent modulation of response strength at these sites is not call-specific. The other type, found in secondary auditory areas (Figures 4 and 6), is variable in its latency from call to call and is not obligatory, i.e. at fast rates they often do not occur. Delivery-dependent modulation of response strength at these sites is call-specific. Even though response strengths for both responses decrease at faster delivery rates, labeling these processes as ‘adaptation’ would suggest a similarity in their causal mechanisms that seems unwarranted.

Some cautionary points should be taken into account in the interpretation of the results. First, our recordings have been made in anesthetized animals. This allows us to conclude that the distinction between common and rare calls is an automatic process, but limits the applicability to understand how auditory systems operate in natural settings. Second, we based the stimulus rates on observed distributions of call recurrence intervals in spontaneous communication, but how natural is it that specific calls recur at such rates 900 times in a row? We queried our behavioral recordings to answer this question, and found that sequences of 900 short-range contact calls by the same animal have a typical mean interval of 1.4 s (mode based on all intervals of all birds, excluding sequences that span different days). We also found that sequences of 900 specific calls with mean recurrence intervals of 5000, 2500, 1250, and 625 ms occur, while 313 ms should be considered a limiting case and 156 ms does not occur. However, we did not apply natural variation in timing from call to call. The effects of such variation on stimulus history-dependent response modulation needs to be addressed in future studies.

Taken together, our results suggest that zebra finch auditory systems are efficient in processing natural scenes that are rife with calls by exploiting the redundancy in sequences of recurring signals over short time-scales. Previous studies on long-term auditory memory in zebra finches have shown that repetition of complex sounds leads to a long-lasting, stimulus-specific decrease of neural responses in NCM [45]. Zebra finches may thus serve as a suitable system to investigate how neural plasticity and memory operate and interact at different time-scales in perceiving patterns of vocalization delivery in natural communication.

## Materials and Methods

### Animals

We used five adult (>120 days old) male and five adult female zebra finches for behavioral recordings, and 12 adult female zebra finches for electrophysiological recordings. All birds had been reared in a colony and subsequently housed in an aviary with other adult zebra finches of both sexes. Prior to the neurophysiological recordings sessions, none of the animals had ever been exposed to the calls used as stimuli, or any other vocalization of the animals from which they originate. All experiments were carried out in accordance with German laws and regulations on animal experiments and were approved by the Government of Upper Bavaria, according to the Tierschutzgesetz, approval number 55.2-1-54-2531-37-06.

### Behavioral Recording

Male and female birds were randomly selected to form five pairs, and were housed in a wire-mesh cage (55×30×34 cm) inside a sound-isolated recording box. The cage was divided into two compartments by wire-mesh that separated the birds but allowed them to freely communicate with each other. Each compartment had its own microphone (4190, Brüel & Kjaer, Bremen, Germany) from which we continuously recorded sound at 16-bit resolution and 44.1 KHz sampling rate (sound card: M-audio delta 44, Hallbergmoos, Germany) on a personal computer running GNU/Linux, with a kernel that allowed for real-time scheduling. Digital signals were saved to disk with a custom-written application based on JACK (http://jackaudio.org). We extracted potential vocalizations from the on-disk recordings by applying a Short-Time Fourier Transform [46] (STFT; 2.9 ms Gaussian window, 0.7 ms step size) and identifying sound episodes, termed ‘notes’, in which energy in at least one of the two channels was above background level in a frequency band from 0.4–10 kHz for at least 15 ms and that did not contain subthreshold gaps larger than 7.5 ms. Notes were extracted with 50-ms margins and stored in a HDF5 file [47] together with information on their duration and time of occurrence.

### Behavioral analyses

For each stored note we determined which of the two recording channels had the largest sound power and used this channel to calculate at 1-ms intervals the fundamental frequency, using an autocorrelation algorithm [48], and the frequency spectrum (12 ms Gaussian window), using a Fast Fourier Transform. From these we calculated per note the first four statistical moments of the 1) fundamental frequency, 2) wiener entropy [49] (a measure for spectral flatness), 3) median frequency, and 4) acoustic power. The results were stored in the same HDF5 file that contained the sounds, together with a spectrogram of each note (0–11 kHz, 128 frequency bins, 500 time bins/s) to be used for later visual verification of the note type. The note sounds, their calculated acoustic features and their spectrographic representations could be queried interactively with arbitrarily complex selection criteria, with a custom-written module (freely available from GJLB upon request) in the scientific programming environment SciPy [50].

Next, we identified short-range contact calls produced by each individual among the total pool of note sounds in the recorded database. We operationally define a short-range contact call as belonging to the call type that is produced most frequently by zebra finches in a social setting. In all recordings that we have analyzed so far (including the 10 animals of the current study) there is only one call type that individual birds produce more or less continuously. Zebra finches do have other call types in their repertoire, but these are produced much less frequently and are easily distinguished from short-range contact calls in long-term recordings. Zann [10] uses the term ‘tet-call’ for those communication sounds that are produced as a constant ‘background’ by wild zebra finches. However, he also mentions that these calls are approximately 50 ms in duration and are chevron-shaped, which in our laboratory population is sometimes, but certainly not always, the case. For this reason we avoid terminology that is linked to bioacoustic structure, and instead simply use ‘short-range contact call’ for those calls that appear to function as such.

Short-range contact calls were distinguished from other sounds in a two-step procedure. First we plotted the fundamental frequency of each note against its duration; male and female short-range contact calls often show distributions that are largely separate from each other and from other vocalizations in this two-dimensional space. In our five pairs of birds this turned out to be the case. Putative sets of male and female calls were selected on the basis of these distributions. Subsequently, sets were cleaned by sorting notes on the remaining acoustic features, visualizing them on-screen using the stored spectrograms, and then removing notes that were not short-range contact calls in the first and last 5% of the sorted set. Using this procedure, we were able to select sets of male and female calls containing a very low number of type I errors, i.e. notes that were classified as a short-range contact call but were in fact a different sound. We estimated the type I error level by selecting a random sample of 2000 calls for each set, and examining them visually one by one to see if their classification was correct. The type I error level was on average 0.25% (range: 0.0%–0.62%). We also estimated the type II error level of our procedure, by examining a random sample of 2000 excluded notes per bird pair and counting the number of missed short-range contact calls it contained. Calls may be missed in our selection procedure because they overlap in time with other vocalizations or with non-vocal sounds like wing fluttering. The type II error level was on average 5.49% (range: 4.82%–6.91%). We did not attempt to distinguish between male and female calls in the type II error category. Assuming they occurred in a similar ratio as in the correctly selected sets, we estimated the error made in the calculation of median call recurrence intervals (see Results) by randomly removing notes in proportion to the type II errors, and subsequently re-calculating this value, leading to a mean decrease of 4.81% (range: 2.28%–7.66%). The reported values are not corrected for these relatively small estimated differences.

### Electrophysiology

We recorded neural activity from 32 different sites in parallel using silicon-based electrode arrays [27], [28] (a4x8-5 mm200-400-413, NeuroNexus Technologies, Ann Arbor, MI). The probes consisted of four parallel shanks separated 400 µm apart, each of which had 8 recording sites separated 200 µm apart. The matrix of 8×4 recording sites thus covered a rectangular plane of 1400×1200 µm in the auditory forebrain (Figure 2). We were primarily interested in activity at the level of local populations of neurons, rather than single cells, and therefore used probes with electrode site areas of 413 µm^2^, which we considered optimal for local-field and multi-unit action potential measurements. This precluded the reliable sorting of multi-unit signals into single-unit spiking activity.

### Acute recording

Birds were anesthetized with isoflurane gas (in oxygen; induction: 3%, maintenance: 1.5%) that flowed through a small mask over the bird's beak. After induction of anesthesia, the bird's head was fixed in a custom-made stereotaxic frame that allowed for sound to reach the ears binaurally. In 10 birds, the beak angle with respect to the shanks of the electrode array was 45°, and in two it was 70°. The bird's body was resting on a heating pad that maintained body temperature during surgery and recording. Lidocaine cream (2%) was applied to the skin overlying the skull and a midline incision was made. A rectangular window was made in the skull to expose both the area over the auditory forebrain where the electrode array would be inserted and the branch point of the midsagittal sinus, which acted as the reference coordinate. After making an incision in the dura the probe was positioned parasagittally with a micromanipulator. The middle shanks were centered at 0.6–0.8 mm lateral and 0.8–1.3 mm rostral from the bifurcation of the midsagittal sinus. Precise positioning depended on the avoidance of blood vessels on top of the brain. The probe was lowered slowly and in small steps until the deepest shank reached a depth of 2500 µm below brain surface. In half of the birds we sampled from the right hemisphere, and in the other half from the left hemisphere. The probe placement was targeted to include field L, but also included parts of NCM and CMM [34], depending on the exact location of the probe in the rostral – caudal direction. Precise positioning depended exclusively on predetermined stereotaxical coordinates and blood vessel avoidance; no search stimuli were used. The electrophysiological recordings presented in this paper are therefore not biased to any response characteristics *a priori* [51]. Playback of stimuli and recording started 30 minutes after the probe was in place.

The 32 electrophysiology channels, referenced to a silver wire under the scalp, were buffered by a headstage preamplifier (10× gain; MPA32I, Multichannel Systems, Reutlingen, Germany) prior to amplification with a multichannel amplifier (250× gain, fixed band-pass filters 0.1–5000 Hz; PGA64, Multichannel Systems). The amplified and filtered signals were then digitized (at 14 kHz, 16 bits resolution; NI9205, National Instruments, Munich, Germany), and stored on a personal computer. We recorded call stimuli that were presented during the experiments with a microphone, and digitized this acoustic signal on the same data-acquisition system as an additional channel. This enabled us to verify the alignment of neural responses with the stimuli with high precision.

### Experimental design

We played six sequences of 1000 call stimuli to each bird. The rate at which calls were delivered was constant within a sequence, but differed between sequences, with the different mean durations from call onset to the next call onset being 5000, 2500, 1250, 625, 313, and 156 ms. As the 1000-call sequence contained 100 rare calls and 900 common calls, the actual average recurrence interval is 5556, 2778, 1389, 694, 347, and 174 ms for common calls, and 50000, 25000, 12500, 6250, 3125, and 1563 ms for rare calls. The exact timing from call to call varied randomly with a maximum of 10 ms around the mean (uniform distribution), enabling us to conclude that highly stimulus-locked neuronal responses were caused by the current call and not by long-latency responses to preceding calls.

To test for stimulus-specific effects, each rate sequence was constructed using two different calls that occurred with different probabilities: one call was given 900 times (‘common’), while the other one was given 100 times (‘rare’). The two call variants were randomly intermixed, so that for each call presentation there was a probability of 0.9 for the common and 0.1 for the rare call to occur.

To prevent call-specific memory effects of calls in earlier blocks on responses to calls in later sequences, we used different common–rare pairs in each of the six rate sequences that an individual bird received (A/B, C/D, E/F, G/H, I/J, K/L; see Figure 3). Different birds received the same six common–rare call pairs, but specific call pairs were assigned to different delivery rate sequences and the role of common and rare occurrence of calls within call pairs was reversed in half of the birds, thereby balancing call delivery across birds. The presentation order of the six rate sequences was randomized for each bird.

### Stimuli

Each of 12 different call variants used in this study (Figure 3) originates from a novel male zebra finch. We selected the six common–rare pairs from a large database containing call recordings from our laboratory, with the only criterion that call variants within pairs had the same duration, but differed in mean fundamental frequency sufficiently to be easily distinguishable by human listeners (see caption Figure 3 for parameters). Other acoustic features differed between the calls as well, but were not selected for. Call amplitudes were scaled so that their average level was 63 dB SPL at the position of the head of the bird and were measured with a microphone (4190, Brüel & Kjaer, Bremen, Germany) that was calibrated before each recording session (4231, Brüel & Kjaer). The playback speaker (Vifa 10 BGS 119/8, Acoustic Systems Engineering, Germany) covered the entire perceptible frequency range of zebra finch vocalizations with one mid-range transducer (∼0.4–10 kHz) and was positioned 0.5 m in front of the animal.

### Data analyses

Digitized electrophysiological recordings were filtered off-line to yield a low-frequency signal (0.1–350 Hz) containing local field potentials (LFP) and a high-frequency signal (0.5–5 kHz) containing analog multi-unit action potentials (AMUA). The multi-unit signal was rectified, averaged into 2.5-ms time-bins, and values below a fixed threshold per site were set to zero so that background signal was excluded while retaining local spiking activity. The threshold level was five median absolute deviations [52] above the median of all samples per bird and was fixed across all sites and all call sequences within birds. From both signals we extracted stimulus-aligned epochs that ranged from 20 ms before call onset to 20 ms before the next call onset. This resulted in a total of 4,608,000 (72,000 calls ×32 channels ×2 signal types) event-related response epochs that formed the bases for our analyses. All data was stored in HDF5 format (version 1.8, http://www.hdfgroup.org) using PyTables 2.0 [47] and analyses were carried out in the scientific computing environment SciPy [50].

We checked for recording artifacts by visually screening all LFP response epochs, and identifying events that showed extreme and long deviations from normal response patterns, leading us to exclude 440 call events (0.6% of the total events). We checked if sites were responsive to auditory stimulation by performing a paired *t*-test (two-tailed, *p*<0.05) on the level of AMUA responses over a 500 ms interval just before and just after the start of calls in the 5000 ms sequence. Twelve sites (out of 384, in four birds) did not show significant auditory AMUA activity associated with call events; histology showed these to be situated in the hippocampus or the ventricle overlying the auditory forebrain (white sites in Figure 5). These sites were excluded from the AMUA analyses but not from the LFP analyses as these signals clearly showed auditory responses originating from the adjacent caudal nidopallium.

To statistically compare the response strength to calls that had different probabilities (common/rare) or occurred in different rate sequences, we calculated the mean of the AMUA signal and the mean power of the LFP signal for individual call events at each site during a response episode of 293 ms (see Results) from the onset of the call. Absolute response levels of individual sites cannot be compared in a meaningful way between birds because of variation in probe placement. To get an overall measure of absolute auditory response strength to each specific call event we therefore averaged the concurrent responses at different sites within an individual, and used this measure as a basis for statistical tests.

### Mapping to anatomy

Prior to implantation, the silicon electrode array probes were coated with the fluorescent dye DiI (probe dipped in DiI solution in DMSO, then dried) in half of the birds for later anatomical registration with histological sections [27], [28]. We did not observe any differences between the electrophysiological signals of birds that had DiI-coated probes and birds that had bare probes. At the end of the experiment, the level of anesthetic was increased to 5% to euthanize the animals, after which the brain was removed and frozen for histology. Nissl staining of sections and fluorescence microscopy were used to verify probe location. Tracts without DiI could rarely be seen in Nissl-stained sections, indicating that the 15 µm thick shanks inflicted little damage to neural tissue, as has been reported for cortical tissue in cats [28]. The tracts of DiI-labeled shanks, however, could easily be detected with fluorescence microscopy.

The auditory forebrain in zebra finches is a relatively large area consisting of Field L, caudomedial nidopallium (NCM) and caudal mesopallium (CM) [34]. The anatomy of Field L in zebra finches has been studied in detail in males [11], [34], and consists of different areas: L, L2, L1, and L3. We are not aware of any study specifically investigating the anatomy of the auditory forebrain in female zebra finches, but there are to our knowledge no indications that the anatomy of the auditory system would be sexually dimorphic. Nevertheless, this is not key to the current study, as we based our anatomical mapping of the electrode array on its position relative to the subarea L2 (see results), which is morphologically well-defined and readily visible in Nissl-sections of female birds [11].

### Statistics

Data were statistically analyzed using open source software R [53], version 2.9.2. We tested the effects of calling rate and call probability (common/rare) on response strength in a linear mixed model with REML estimation, using the LMER procedure of the lme4 library [54] with *calling rate* and *probability* and their interaction as fixed factors and *bird* as a random factor. LMER provides a model of the observed data that can be evaluated for goodness of fit. We used mean LFP and AMUA response strengths as dependent variables, which were obtained for each bird and treatment level by averaging over the repeated measurements taken during the trial. Mean response strength values were log-transformed in order to meet the standard assumptions of normality of residuals and homogeneity of variances. In the case of AMUA responses we calculated two separate response means, one for sites that had stereotypic and stimulus-locked responses (mean correlation coefficient between responses >0.5, see results). This data set was fitted with an extra fixed factor, *site type*, that distinguished between these two groups, but otherwise was identical to the LFP model. Comparisons are reported as *t*-statistics, with significance values computed using Monte Carlo Markov chain sampling with 50,000 iterations, as calculated by the pvals.fnc function of the languageR library [54]. It should be noted that the mixed-model statistical analysis of neural responses depends solely on mean local firing rates or the mean local field potential power and ignores potential stimulus-dependent information that may be present in systematically different patterns of activity.

## References

[1] Barlow H, Rosenblith W (1961). Possible principles underlying the transformations of sensory messages.. Sensory Communication.

[2] Müller JR, Metha AB, Krauskopf J, Lennie P (1999). Rapid adaptation in visual cortex to the structure of images.. Science.

[3] Ulanovsky N, Las L, Nelken I (2003). Processing of low-probability sounds by cortical neurons.. Nat Neurosci.

[4] Näätänen R, Tervaniemi M, Sussman E, Paavilainen P, Winkler I (2001). ‘Primitive intelligence’ in the auditory cortex.. Trends Neurosci.

[5] Nelken I, Ulanovsky N (2007). Mismatch negativity and stimulus-specific adaptation in animal models.. J Psychophysiol.

[6] Szymanski FD, Garcia-Lazaro JA, Schnupp JWH (2009). Current source density profiles of stimulus-specific adaptation in rat auditory cortex.. J Neurophysiol.

[7] von der Behrens W, Bauerle P, Kossl M, Gaese BH (2009). Correlating stimulus-specific adaptation of cortical neurons and Local Field Potentials in the awake rat.. J Neurosci.

[8] Ulanovsky N, Las L, Farkas D, Nelken I (2004). Multiple time scales of adaptation in auditory cortex neurons.. J Neurosci.

[9] Ribler R, Marthur A, Abrams M (1996). Visualizing and modeling categorical time series data..

[10] Zann RA (1996). The Zebra Finch..

[11] Fortune ES, Margoliash D (1992). Cytoarchitectonic organization and morphology of cells of the field L complex in male zebra finches (Taenopygia guttata).. J Comp Neurol.

[12] Fortune ES, Margoliash D (1995). Parallel pathways and convergence onto HVc and adjacent neostriatum of adult zebra finches (Taeniopygia guttata).. J Comp Neurol.

[13] Wild JM, Karten HJ, Frost BJ (1993). Connections of the auditory forebrain in the pigeon (Columba livia).. J Comp Neurol.

[14] Bolhuis JJ, Gahr M (2006). Neural mechanisms of birdsong memory.. Nat Rev Neurosci.

[15] Gehr DD, Capsius B, Gräbner P, Gahr M, Leppelsack HJ (1999). Functional organisation of the field-L-complex of adult male zebra finches.. Neuroreport.

[16] Cousillas H, Leppelsack H, Leppelsack E, Richard J, Mathelier M (2005). Functional organization of the forebrain auditory centres of the European starling: A study based on natural sounds.. Hearing Res.

[17] Grace JA, Amin N, Singh NC, Theunissen FE (2003). Selectivity for conspecific song in the zebra finch auditory forebrain.. J Neurophysiol.

[18] Lewicki MS, Arthur BJ (1996). Hierarchical Organization of Auditory Temporal Context Sensitivity.. J Neurosci.

[19] Müller CM, Leppelsack HJ (1985). Feature extraction and tonotopic organization in the avian auditory forebrain.. Exp Brain Res.

[20] Nagel KI, Doupe AJ (2008). Organizing principles of spectro-temporal encoding in the avian primary Auditory Area Field L.. Neuron.

[21] Sen K, Theunissen FE, Doupe AJ (2001). Feature analysis of natural sounds in the songbird auditory forebrain.. J Neurophysiol.

[22] Theunissen FE, Sen K, Doupe AJ (2000). Spectral-temporal receptive fields of nonlinear auditory neurons obtained using natural sounds.. J Neurosci.

[23] Gill P, Woolley SMN, Fremouw T, Theunissen FE (2008). What's that sound? Auditory area CLM encodes stimulus surprise, not intensity or intensity changes.. J Neurophysiol.

[24] Amin N, Doupe A, Theunissen FE (2007). Development of selectivity for natural sounds in the songbird auditory forebrain.. J Neurophysiol.

[25] Boumans T, Theunissen FE, Poirier C, Linden AVD (2007). Neural representation of spectral and temporal features of song in the auditory forebrain of zebra finches as revealed by functional MRI.. Eur J Neurosci.

[26] Nagel KI, Doupe AJ (2006). Temporal processing and adaptation in the songbird auditory forebrain.. Neuron.

[27] Csicsvari J, Henze DA, Jamieson B, Harris KD, Sirota A (2003). Massively parallel recording of unit and Local Field Potentials with silicon-based electrodes.. J Neurophysiol.

[28] Blanche TJ, Spacek MA, Hetke JF, Swindale NV (2005). Polytrodes: high-density silicon electrode arrays for large-scale multiunit recording.. J Neurophysiol.

[29] Abeles M (1982). Local cortical circuits: An electrophysiological study..

[30] Destexhe A, Contreras D, Steriade M (1999). Spatiotemporal analysis of Local Field Potentials and unit discharges in cat cerebral cortex during natural wake and sleep states.. J Neurosci.

[31] Terleph TA, Mello CV, Vicario DS (2006). Auditory topography and temporal response dynamics of canary caudal telencephalon.. J Neurobiol.

[32] Squires K, Wickens C, Squires N, Donchin E (1976). The effect of stimulus sequence on the waveform of the cortical event-related potential.. Science.

[33] Marler P, Marler P, Slabbekoorn H (2004). Bird calls: a cornucopia for communication.. Nature's Music.

[34] Vates GE, Broome BM, Mello CV, Nottebohm F (1996). Auditory pathways of caudal telencephalon and their relation to the song system of adult male zebra finches (Taenopygia guttata).. J Comp Neurol.

[35] Grill-Spector K, Henson R, Martin A (2006). Repetition and the brain: neural models of stimulus-specific effects.. Trends Cogn Sci.

[36] Condon CD, Weinberger NM (1991). Habituation produces frequency-specific plasticity of receptive fields in the auditory cortex.. Behav Neurosci.

[37] Calford MB, Semple MN (1995). Monaural inhibition in cat auditory cortex.. J Neurophysiol.

[38] Brosch M, Schreiner CE (1997). Time course of forward masking tuning curves in cat primary auditory cortex.. J Neurophysiol.

[39] Malone BJ, Semple MN (2001). Effects of auditory stimulus context on the representation of frequency in the gerbil inferior colliculus.. J Neurophysiol.

[40] Friston K (2005). A theory of cortical responses.. Phil Trans R Soc B.

[41] May P, Tiitinen H, Ilmoniemi RJ, Nyman G, Taylor JG (1999). Frequency change detection in human auditory cortex.. J Comput Neurosci.

[42] Jääskeläinen IP, Ahveninen J, Bonmassar G, Dale AM, Ilmoniemi RJ (2004). Human posterior auditory cortex gates novel sounds to consciousness.. Proc Natl Acad Sci USA.

[43] Winkler I (2007). Interpreting the mismatch negativity.. J Psychophysiol.

[44] Garrido MI, Friston KJ, Kiebel SJ, Stephan KE, Baldeweg T (2008). The functional anatomy of the MMN: A DCM study of the roving paradigm.. NeuroImage.

[45] Chew SJ, Vicario DS, Nottebohm F (1996). A large-capacity memory system that recognizes the calls and songs of individual birds.. Proc Natl Acad Sci U S A.

[46] Cohen L (1995). Time-frequency analysis: theory and applications..

[47] Alted F, Vilata I, Prater S, Mas V, Hedley T (2002). PyTables: Hierarchical datasets in Python.. http://www.pytables.org/.

[48] Boersma P (1993). Accurate short-term analysis of the fundamental frequency and the harmonics-to-noise ratio of a sampled sound.. Proceedings of the Institute of Phonetic Sciences, Amsterdam.

[49] Tchernichovski O, Nottebohm F, Ho CE, Pesaran B, Mitra PP (2000). A procedure for an automated measurement of song similarity.. Anim Behav.

[50] Jones E, Oliphant T, Peterson P, others (2001). SciPy: Open source scientific tools for Python.. http://www.scipy.org/.

[51] Kriegeskorte N, Simmons WK, Bellgowan PSF, Baker CI (2009). Circular analysis in systems neuroscience: the dangers of double dipping.. Nat Neurosci.

[52] Venables W, Ripley B (1999). Modern applied statistics with S-PLUS..

[53] R Development Core Team (2009). R: A Language and environment for statistical computing.. http://www.R-project.org.

[54] Baayen R, Davidson D, Bates D (2008). Mixed-effects modeling with crossed random effects for subjects and items.. J Mem Lang.

[55] Gardner TJ, Magnasco MO (2006). Sparse time-frequency representations.. Proc Natl Acad.Sci USA.

